# Newly Developed Self-Assembling Antioxidants as Potential Therapeutics for the Cancers

**DOI:** 10.3390/jpm11020092

**Published:** 2021-02-02

**Authors:** Babita Shashni, Yukio Nagasaki

**Affiliations:** 1 Department of Materials Science, Graduate School of Pure and Applied Sciences, University of Tsukuba, Tennoudai 1-1-1, Tsukuba, Ibaraki 305-8573, Japan; shashni@ims.tsukuba.ac.jp; 2 Master’s School of Medical Sciences, Graduate School of Comprehensive Human Sciences, University of Tsukuba, Tennoudai 1-1-1, Tsukuba, Ibaraki 305-8573, Japan; 3 Center for Research in Isotopes and Environmental Dynamics (CRiED), University of Tsukuba, Tennoudai 1-1-1, Tsukuba, Ibaraki 305-8573, Japan

**Keywords:** cancer, reactive oxygen species, antioxidant, self-assembling drug

## Abstract

Elevated reactive oxygen species (ROS) have been implicated as significant for cancer survival by functioning as oncogene activators and secondary messengers. Hence, the attenuation of ROS-signaling pathways in cancer by antioxidants seems a suitable therapeutic regime for targeting cancers. Low molecular weight (LMW) antioxidants such as 2,2,6,6-tetramethylpyperidine-1-oxyl (TEMPO), although they are catalytically effective in vitro, exerts off-target effects in vivo due to their size, thus, limiting their clinical use. Here, we discuss the superior impacts of our TEMPO radical-conjugated self-assembling antioxidant nanoparticle (RNP) compared to the LMW counterpart in terms of pharmacokinetics, therapeutic effect, and adverse effects in various cancer models.

## 1. Introduction

Reactive oxygen species (ROS) are intracellular free oxygen radicals with one or more unpaired electrons in their valency shell. These unpaired electrons are capable of independent existence and are highly reactive, who tend to stabilize their shell by donating or extracting electron(s) from the oxidizable molecules. These target oxidizable molecules become a radical entity, which further starts a chain reaction of damaging other molecules [1]. The concept of organic free radical began in 1900 by Gomberg, who speculated the presence of triphenyl methyl radical (Ph3C^•^) in the living system. In 1954, a free radical theory was proposed by Gershman, who pointed out the toxicity of oxygen and its reduced forms due to the highly oxidizing power [2,3]. In 1969, McCord and Fridovich discovered the first cellular antioxidant enzyme, superoxide dismutase (SOD) [4].

ROS are broadly classified into radical and non-radical species. Radical species involve entities with unpaired electron(s) such as superoxide (O_2_^●−^), hydroxyl radical (OH^●−^), oxygen biradicals (O_2_^••^), peroxyl radicals (ROO^•^), and alkoxy-radicals (RO^•^). In contrast, non-radical species include entities that do not contain an unpaired electron but can easily convert to free radicals in the living system. The primary reported species are hydrogen peroxide (H_2_O_2_), hypochlorous acid (HOCl), ozone (O_3_), singlet oxygen (^1^O_2_), organic peroxides (ROOH), aldehydes (RCHO), and so on [5]. ROS can be produced both through endogenous and exogenous sources. Endogenous sources of ROS are mitochondria, peroxisomes, endoplasmic reticulum, and activated inflammatory neutrophils. Large amount of ROS is generated in mitochondria via several enzymatic reactions such as an electron transport chain, NADH dehydrogenase, and ubiquinone cytochrome C reductase, etc. Several enzymatic reactions generate ROS in peroxisomes (β-oxidation of fatty acids; acyl CoA oxidase, uric acid metabolism; urate oxidase, xanthine metabolism; xanthine oxidase, D-proline metabolism; D-amino acid oxidase), and in the endoplasmic reticulum (cytochrome P450, b5 enzymes, diamine oxidase, thiol oxidase enzyme Erop1p). Activated inflammatory cells such as neutrophils also produce numerous ROS in the inflammatory sites.

In contrast, exogenous sources include pesticides, ultraviolet light, air, and water pollution, metals such as iron, copper, cobalt, cadmium, arsenic, etc. [6,7]. Under normal conditions, a small amount of ROS escapes during the intracellular processes regulated by the enzymatic antioxidant system, viz., superoxide dismutase, catalase, peroxiredoxins, glutathione peroxidases, glutathiones, bilirubin, etc. [6]. Although antioxidant systems maintain a tightly controlled redox homeostasis in the normal cells, irreversible or non-repairable oxidative damage to nuclear and mitochondrial DNA, protein, and lipids are inevitable due to their prolonged overexposure to exogenous ROS producers. These overproduced ROS lead to oxidative stress-related diseases such as cancer, cardiovascular diseases, diabetes, rheumatoid arthritis, neurogenerative diseases, liver disease, and ischemic and post-ischemic pathologies [6,7,8]. Exogenous oxidative stress or prolonged chronic endogenous oxidative stress such as inflammation has been linked to tumor initiation, promotion and progression, which are evident from the fact that cancer cells are under constant oxidative stress, a hallmark of cancerous phenotype [8,9]. Considering a continuous elevated ROS level in the tumor environment, which is crucial for tumorigenesis, metastasis, and angiogenesis, antioxidant therapies seem to be the most intuitive and apt intervention to attenuate various cancers. Although various low molecular weight (LMW) antioxidants such as vitamin C, vitamin E, selenium, and TEMPOL, showed effectiveness in vitro and in some cases in vivo; however, clinically, they failed to show any conclusive efficacy [10]. Their clinical failure may be attributed to their metabolism and rapid excretion, preventing them from reaching the target ROS production site of the tumor cells in enough amount to scavenge ROS to a critical level to have sufficient anti-cancer efficacy. Another significant and severe problem of the LMW antioxidants is that they internalize in the healthy cells and disturb their redox homeostasis, including the mitochondrial electron transport chain. Here, we conceptualized new antioxidants, “self-assembling antioxidants”, which significantly vary in their pharmacokinetic characteristics and reduce undesired adverse side effects related to the LMW antioxidants. In this review, an implication of ROS in cancer, the status of antioxidant cancer therapies using LMW compounds and the precedence of self-assembling antioxidants (we abbreviate them as redox nanoparticle hereafter; RNP) over LMW antioxidant compounds for the cancer therapeutics will be discussed in detail.

## 2. Oxidative Stress and Cancer

As described in the above section, evidence from the clinical and bench studies indicate that the elevated intracellular ROS contributes to cancer initiation, promotion and progression [8,9]. The intracellular antioxidant system can quench the overproduced ROS generated through the exogenous source or chronic inflammation in the normal cells to some extent and under their capacity. However, ROS that could not be completely eliminated could be mutagenic and induce carcinogenesis [9,11,12]. For instance, white blood cells convert to neutrophils and invade the inflamed colon in ulcerative colitis. These activated neutrophils generate ROS such as O_2_^●−^ and HOCl, which are known to stimulate mutagenesis and cause colon cancer [13,14]. Similarly, constant exposure to free radical producers such as ultraviolet, tobacco smoke, and metal ions may stimulate mutagenesis and induce melanoma, bronchogenic carcinoma, and colorectal cancer, respectively [8].

Tumor initiation is triggered by damaging cellular genes, mainly by the oxidation with ROS. It is reported that about 10,000 oxidative hits to DNA per cell are observed daily in humans [15], which are eventually recovered by the cellular repairing system. However, sometimes, when oxidative stress damage is beyond their repair capacity, DNA base adduct with non-scavenged ROS may be observed [8,16]. For instance, one of ROS, hydroxide radical (•OH) attacks the guanine (G) base at the eighth position to become 8-OH-G, which leads to Guanine-Cytosine to Thymine-Adenine transversion, called point mutation [8,16,17]. In addition, DNA helix alterations such as single or double-strand breaks and inter-strand crosslinks are also observed upon damage by free radicals generated through ultraviolet or ionizing lights [18,19]. Such alteration in the DNA results in genomic instability, which may further modulate transcription and transduction pathways favoring carcinogenesis and tumor progression [20,21].

ROS in tumors participates in the intracellular signaling and regulation by acting as secondary messengers [6,7,8]. Ras protein family, one of the membrane-bound G protein families, regulates transcription, cell growth, and apoptosis [22]. Ras is activated by ROS derived from ultraviolet radiation and metal ions and is known to be frequently mutated in humans cancers such as skin, liver, and colon cancers [22]. It should be noted that Ras-dependent cell proliferation requires ROS, which is unconditionally elevated in cancers [23].

Another tumor suppressor protein, p53, a transcriptional factor, is known to be involved in cell cycle arrest, senescence, apoptosis, DNA repair, and redox homeostasis [24,25,26]. Upon oxidative stress by ionizing radiation or genotoxic insults, DNA lesions are accumulated, which are repaired before the DNA replication by arresting the cell cycle. Once the DNA lesion is repaired, the normal cell resumes cell division. p53, known as “the guardian of the genome”, preserves this DNA integrity [27]. However, when the TP53 gene is mutated, the DNA damage is carried down to several cell divisions, leading to chromosomal rearrangement [28]. TP53 gene is often known to be mutated in various solid cancers [27].

Another popular redox-sensitive transcription factor is NF-kB, which is reported to be involved in cell survival, differentiation, growth, angiogenesis, and inflammation [29,30]. NF-kB is activated by carcinogenic stimuli such as ultraviolet radiation, phorbol esters, toxic metals, and asbestos, all of which are oxidative stress inducers [31]. Although it is evident from several reports that ROS activates NF-kB, recent studies confirm the bidirectional regulation by ROS, which is not clearly understood [30]. Nonetheless, it is reported that the NF-kB pathway is often excessively activated in tumor tissues, promoting tumor cell proliferation and survival [32].

It is reported that ROS also activates protein kinases C (PKCs), critical for cancer proliferation, by increasing the cytosolic calcium concentration and the cysteine oxidization of their regulatory domains [33,34]. This activates downstream cell proliferation, differentiation, and apoptosis pathways, involving mitogen-activated protein kinases (MAPKs) such as extracellular-regulated (ERKs), c-jun-NH_2_-terminal kinase (JNKs), and p38 MAPK [35]. Furthermore, ROS also regulates hypoxia-inducible factor, HIF-1, in tumors, which further modulates many cancer-related genes, such as VEGF, involved in tumor progression and angiogenesis [36]. Other ROS-sensitive regulatory proteins such as AP-1 and nuclear factor of activated T cells are also known to be involved in tumorigenesis [37,38,39]. Interestingly, ROS also regulates pro-proliferative signaling in tumors and prevent apoptosis by activation of proto-oncogene BCL-2, which is an anti-apoptotic protein. BCL-2 family is overexpressed in many cancers such as breast, lung, colorectal, and melanoma, which not only prevents tumor cell death but also promotes their migration, invasion, and metastasis [40].

From the evidence stated above, it is obvious that oxidative stress is critical for tumor initiation and growth by inducing genomic instability and acting as signaling molecules to modulate factors favoring tumorigenesis, angiogenesis, and metastasis, respectively. Since the critical roles of the elevated ROS-signaling pathways are revealed in various cancers, the antioxidant therapies seem to be the most appropriate strategy to impede their growth. The next section will discuss the status of conventional antioxidants for cancer therapy.

## 3. Conventional Antioxidants for Potential Cancer Therapy

As mentioned above, since ROS is strongly associated with carcinogenesis, tumorigenesis, and metastasis, antioxidant treatments to inhibit cancers have been investigated. Sharma et al. reported that patients with locally advanced squamous cell carcinoma of the tongue had significantly elevated plasma lipid peroxidation levels and conjugated dienes. At the same time, primary endogenous antioxidants such as glutathione, vitamin C, vitamin E, glutathione peroxidase, and superoxide dismutase were significantly decreased, as compared to the healthy controls, implying that oxidative stress plays an essential role in the pathophysiology of tongue cancer [41]. Considering the critical role of ROS in tumors, various antioxidants, including natural antioxidants have been tested to dampen the ROS levels as therapeutic interventions. Numerous natural and synthetic antioxidants have been investigated as potential anti-cancer drugs. These investigations have shown positive effects in vitro and/or in vivo against various cancer models. For example, one of the famous synthetic antioxidants, N-acetylcysteine (NAC), has shown the anti-cancer effect on prostate carcinoma, PC-3 cells (in vitro) and human tongue squamous carcinoma, HSC-3 cells (in vivo) [42,43]. Natural vitamins are also reported to exert anti-cancer effects. For instance, vitamin C inhibited invasion and metastasis of breast cancer cells (in vivo) and impaired tumor growth and eradicated liver cancer stem cells in the xenograft model of a hepatocellular carcinoma cell line [44,45]. Vitamin E analog, RRR-α-tocopherol succinate, is known to induce apoptosis mediated death in MDA-MB435, MDA-MB231, and SKBR-3 human breast cancer cells [46,47]. Quercetin, a bioflavonoid, is also known to inhibit cancer growth by arresting the cell cycle and induced apoptosis in breast cancer, prostate cancer and colorectal cancer [48,49,50].

TEMPOL, a redox-cycling nitroxide (4-hydroxy-TEMPO; 4-hydroxy-2,2,6,6-tetramethylpiperidine-1-oxyl), is known as a probe of electron spin resonance due to the presence of unpaired electron in the compound. Since this unpaired spin is stable because of the steric hindrance of the surrounding four methyl groups, so they do not react to each other. However, it is known that TEMPOL can rapidly react with free radicals of ROS. Thus, they can be regarded as one of the most potent antioxidants, like a superoxide dismutase [51]. Luo et al. reported a comparative superoxide inhibition activity of TEMPOL and several other antioxidants in angiotensin II-stimulated preglomerular vascular smooth muscle cells assessed by lucigenin-enhanced chemiluminescence. They confirmed that PEGylated-SOD and TEMPOL exhibited the maximum catalytic actions to scavenge O_2_^●−^ than NAC, vitamin C and E analogues such as ascorbate, α-tocopherol and 6-hydroxy-2,5,7,8-tetramethylkroman-2-carboxy acid (Trolox) and other uncharacterized antioxidants; 5,10,15,20-tetrakis (4-sulphonatophenyl)porphyrinate iron (III)(Fe-TTPS), 2-phenyl-1,2-benzisoselenazol-3(2H)-one (ebselen), nitroblue tetrazolium (NBT) and (−)-*cis*-3,3′,4′,5,7-pentahydroxyflavane (2*R*,3*R*)-2-(3,4-dihydroxyphenyl)-3,4-dihydro-1(2*H*)-benzopyran-3,5,7-triol(-epicatechin) [51,52]. With such high catalytic activity, TEMPOL has been the most preferred choice for antioxidant-based therapy for various oxidative stress-related models such as fibrosis, diabetes, neurodegenerative diseases, radio-protection, ischemia-reperfusion injury and inflammation, hypertension, and cancer [53]. Several studies have demonstrated that TEMPOL inhibits tumor growth and decreases tumor incidence. For instance, TEMPOL induced apoptotic cell death in MDA-MB231 breast cancer cell line [54]. Gariboldi et al. reported the inhibitory effects of TEMPOL on the growth of neoplastic than non-neoplastic cell lines such as breast cancer cell line MCF-7, p53-negative human leukemia cell line HL60, and C6 glioma cells [55,56,57]. Schubert et al. reported that dietary TEMPOL administration to ataxia telangiectasia mutated (ATM)-deficient young mice (develop tumors), prolonged latency to tumors, decreased ROS and oxidative damage, and increased their life span [58]. Corroborating this, Mitchell et al. confirmed that long-term TEMPOL treatment decreased spontaneous tumorigenesis in C3H mice [59]. TEMPO (2,2,6,6-tetramethylpiperidine-1-oxyl) administration into LNCaP tumor-bearing mice also showed significant inhibition to prostate tumor growth [60].

As described above, there are many publications about antioxidant-based cancer chemotherapy. Although numerous antioxidants have been proposed as an efficient anti-cancer agent in vitro and in vivo, these antioxidants failed to show any cumulative effect clinically on healthy, at-risk, and cancer population [10]. For instance, daily supplementation with selenium (200 μg) and/or Vitamin E (400 IU) did not reduce the incidence of prostate or other cancers. Instead, vitamin E supplementation resulted in 17% increase in prostate cancer incidence [61,62]. Corroborating this, daily supplementation of β-carotene (50 mg) also did not reduce the incidence of prostate cancer or other cancers [63]. However, daily supplementation of beta-carotene (15 mg), alpha-tocopherol (30 mg), and selenium (50 μg) to Chinese at-risk population of developing esophageal cancer and gastric cancer reduced cancer mortality associated with gastric cancer, no effect was seen in esophageal cancer suffering population [64]. In another clinical study, a population who were occupationally exposed to asbestos were supplemented with β-carotene (30 mg) and retinyl palmitate (25,000 IU) daily, which tended to associate with increased lung cancer incidence and mortality [65].

Such contrasting effects of conventional antioxidants in tumorigenesis and inconclusive clinical trials indicate that these conventional antioxidants cannot be used for anti-cancer therapy. Because several elegant studies confirmed the role of elevated ROS in cancer, it is striking to see the failure of antioxidant-based cancer therapy. Their clinical failure could be attributed to several factors such as the level and the location of ROS scavenged and the tumor stage at which antioxidants were introduced. In addition, since most conventional antioxidants are LMW, their extremely rapid renal clearance and very low bioavailability may have led to their insufficient accumulation in the tumors resulting in low efficacy.

Another significant problem with the conventional antioxidants is their molecular size-based adverse effects. Mitochondria in the healthy cells generate ATP via an electron transport chain by oxidation of glucose. During this process, a considerable amount of ROS is produced. LMW antioxidants can rapidly spread to the entire body and internalize into the healthy cells, which causes the dysfunction of the essential redox homeostasis, including the electron transport chain, known as “Mithohormesis” [66]. It is reported that treatment with beta carotene, vitamin A, and vitamin E increased mortality in a randomized clinical investigation of more than 230,000 participants [67]. This means that high dose of LMW antioxidants cannot be administered due to their ability to damage mitochondria. Contrarily, the limited dose of the LMW antioxidants may scavenge low ROS sufficiently to stimulate the survival and proliferation of tumor cells rather than impeding it. This was evident in the studies by Gal et al., who reported that administration of NAC and Trolox, Vitamin E analog, increased lymph node metastasis of malignant melanoma [68]. Furthermore, due to the limited dose and poor pharmacokinetic properties, it is also possible that in clinical trials, LMW antioxidants did not reach the target location to quench crucial ROS, e.g., mitochondria of cancer cells. Porporato et al. reported that mitochondrial superoxide promotes migration, invasion, and clonogenicity of tumor cells, which was prevented upon its scavenging [69].

As mentioned above, most antioxidants are small molecules, which contributes to poor bioavailability, prevents target accumulation and causes mitochondrial damage. To overcome these limitations of LMW antioxidants, a delivery platform (nanoparticle) to modulate their pharmacokinetics property has been employed. Nanoparticles-based delivery of antioxidants may scavenge ROS below critical levels in tumors to inhibit their growth due to their higher bioavailability and enhanced permeability and retention (EPR) effects as compared to their LMW counterparts [70]. Several groups have reported the use of antioxidants with various delivery (drug delivery system; DDS) platforms. For example, quercetin-encapsulated liposomes showed in vitro anti-proliferation effect on the breast cancer cells, MCF-7 [71]. Nanoparticles with intrinsic redox ability also showed anti-proliferative and anti-tumor effects, such as mesoporous silica nanoparticles and cerium oxide nanoparticles [72,73]. However, several antioxidant-based delivery platforms have shown practical inhibitory effects in vitro with limited or no in vivo application. Furthermore, silica and cerium oxide have been reported to exert toxicity in mice models with biodegradability issues, thereby limiting their further use [72,74]. One of the major problems with conventional DDS is that the physically encapsulated drug leaks out of the system before reaching their target, which diminishes their efficacy and increases adverse effects. In order to achieve effective antioxidant cancer chemotherapy, a new strategy should be required to overcome the limitation of nanoparticles with physically encapsulated antioxidants. For the last decade, we have devoted a novel designed self-assembling antioxidants to treat oxidative stress-related diseases. The next section will discuss the design, structure, and advantages of our newly developed self-assembling antioxidants for cancer therapy.

## 4. Novel Self-Assembling Antioxidants; Nitroxide Radical-Containing Nanoparticle (RNP)

### 4.1. Design and Structure of RNPs

**Figure 1:**
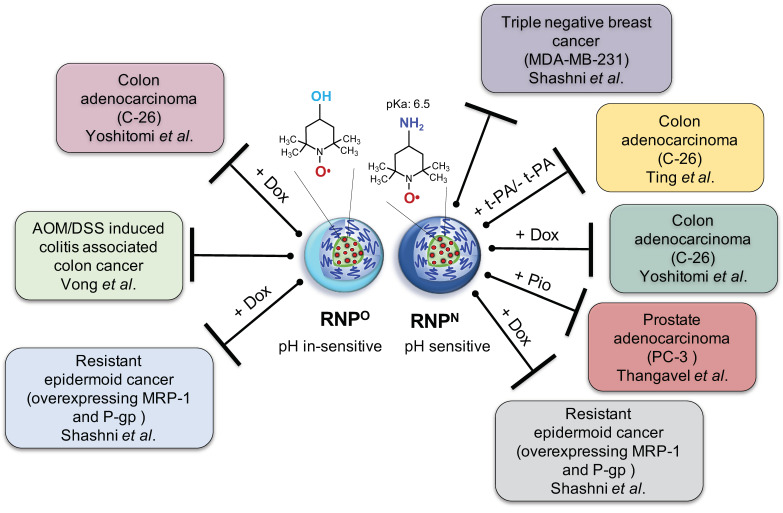
Illustration displaying the therapeutic efficacy of pH sensitive redox nanoparticle (RNP)^N^ and pH in-sensitive RNP^O^ in various cancer models as stand-alone or as adjuvants with conventional anti-cancer drugs; doxorubicin (Dox) and pioglitazone (Pio).

Although TEMPO is one the most potent antioxidants known, similar to the antioxidant enzyme, SOD, its clinical use is greatly limited due to its off-target effects, which can be attributed to its poor pharmacokinetic properties as stated above. In order to improve the pharmacokinetic properties to obtain high efficacy with negligible off-target effects, we have functionalized TEMPO and developed two different types of nitroxide radical-containing nanoparticles (RNPs); pH-sensitive (RNP^N^) and pH-insensitive (RNP^O^), and evaluated their ROS-reduction mediated anti-cancer effect in various in vitro and in vivo models of cancers as stand-alone or as adjuvants to reduce the aggressiveness or sensitize several cancers for the chemotherapy (Figure 1). Since TEMPO possesses an unpaired electron, it is an electron spin resonance (ESR) active species, which could be used for magnetic resonance imaging and pharmacokinetic studies. This property along with its powerful ROS scavenging ability, prompted us to employ TEMPO over other LMW antioxidants for developing self-assembling antioxidants for the biomedical applications [53,75,76].

**Figure 2:**
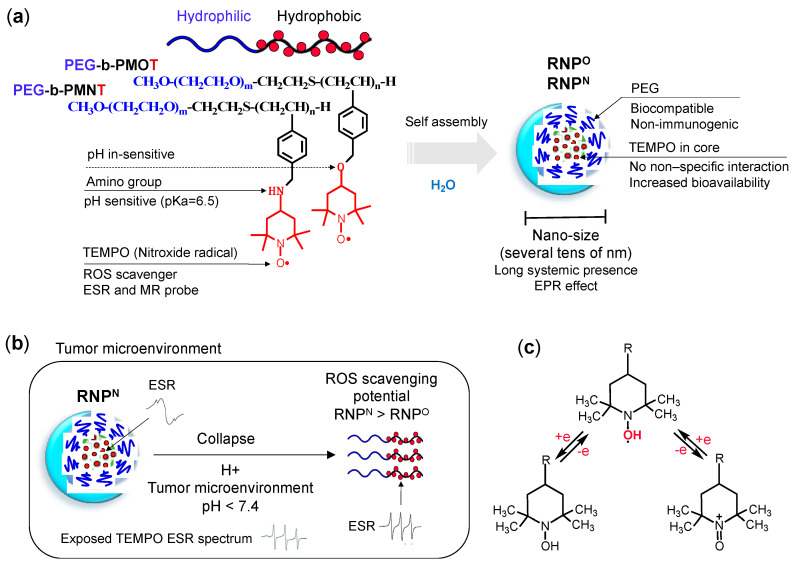
(**a**) Design and structure of antioxidant amphiphilic block copolymers, PEG-*b*-PMOT and PEG-*b*-PMNT, which self-assembles in aqueous media to form nano-sized micelles: pH-insensitive RNP^O^ and pH-sensitive RNP^N^, respectively, used for cancer therapy. (**b**) Illustration showcasing pH-sensitive characteristic of RNP^N^ in the diseased environment (tumor); the amino groups of antioxidant TEMPO moieties in the copolymer (pKa 6.5) is protonated under the low pH in the tumor environment, leading to collapse of RNP^N^ micelle, which enhances its ROS scavenging potential than pH-insensitive RNP^O^. The exposed radical of TEMPO can be detected by Electron Spin Resonance (ESR) as sharp triplet peaks, but when it is in the core of stable RNP^N^, the ESR signal of TEMPO broadens. At low pH, due to disassembly of RNP^N^, TEMPO radical is exposed and displays characteristic sharp triplet peaks of TEMPO. This ESR sensitive characteristic is essential for the pharmacokinetics studies of RNPs. (**c**) Reduction and oxidization reaction equations of TEMPO.

RNPs are comprised of self-assembling amphiphilic block copolymer consisting of a hydrophilic poly (ethylene glycol) (PEG) segment and a hydrophobic poly (chloromethylstyrene) (PCMS) segment (Figure 2a). The chloromethyl groups of the PCMS segment are converted to TEMPO via the substitution of PEG-*b*-PCMS polymer with either 4-amino-TEMPO or with 4-hydroxyl-TEMPO to form base polymers: PEG-*b*-PMNT and PEG-*b*-PMOT, respectively [75,76,77,78]. Under physiological conditions, the block copolymer assembles into a core shell-type micelles with the hydrophobic segment (PMNT or PMOT, which contains TEMPO moieties as a side chain) in the core and hydrophilic PEG in the shell (cumulative average diameter of approximately 20–50 nm).

Since TEMPO moiety conjugates via ether linkage to the PMOT segment, PEG-*b*-PMOT gives a pH-insensitive RNP^O^. In contrast, PEG-*b*-PMNT gives pH-sensitive RNP^N^, because TEMPO conjugates to the PMNT segment via the amine linkage, which protonates under the acidic environment and changes its water solubility. Since the pKa of the amino group of the PMNT segment is ca. 6.5, most of the amino groups in RNP^N^ are not protonated under physiological conditions. However, under the acidic conditions, the protonated amine in the PMNT segment increases, converts their hydrophobic character to the hydrophilic, which weakens the core-coagulation force, leading to the collapse the micelle. Since the inflamed area such as the tumor environment, is known as decreased pH, we anticipated an increase in their antioxidant capacity by the exposure of TEMPO moiety due to collapsed RNP^N^ (Figure 2b,c). This means that both pH-insensitive RNP^O^ and pH-sensitive RNP^N^ will remain intact as a nano-sized self-assembling structure in the blood (pH 7.4). In contrast, under a low pH environment, e.g., cancer, only pH-sensitive RNP^N^ will collapse into individual polymers and show higher ROS scavenging potential than its intact micelle structure and RNP^O^ [78].

Following are the characteristics of RNPs validating its suitability for their use in in vivo applications.

**Figure 3:**
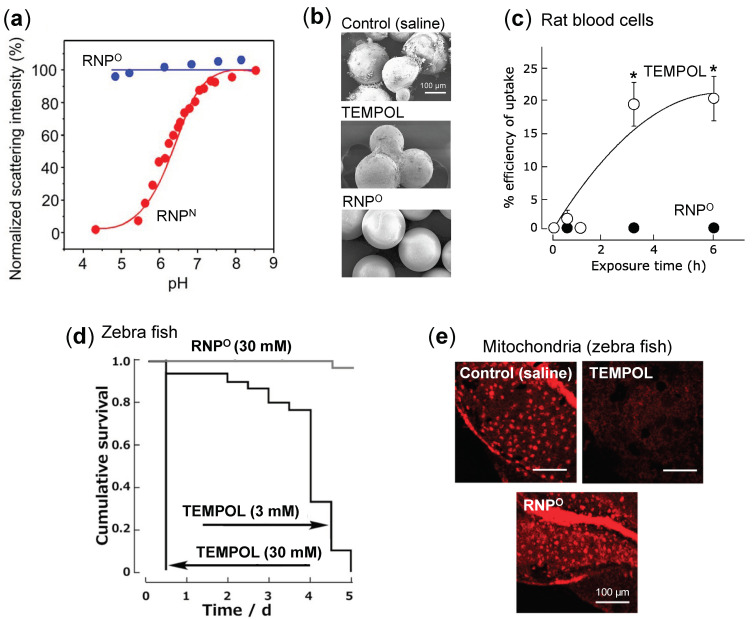
Characterization and non-toxicity of RNPs. (**a**) Laser light scattering intensity of RNP^O^ and RNP^N^ as a function of pH, assessed by dynamic light scattering [77]. (**b**) SEM images of glass beads spiked in rat whole blood with saline, TEMPOL, and RNP^O^ (5 mM) for 30 min [85]. (**c**) The cellular uptake of TEMPOL and RNP^O^ by rat whole blood cells evaluated by ESR [85]. (**d**) Cumulative survival of zebrafish embryo maintained in RNP^O^ (30 mM) and TEMPOL (3 and 30 mM) [88]. (**e**) Microscopic images of the mitochondrial damage in zebrafish larva after 12 h of treatment, assessed by mitotracker and analyzed using a fluorescent confocal microscope system, scale bar 100 μm [88]. * *p* < 0.05 was considered significant. This figure is reproduced with permission from References [77,85,88]. Copyright 2011, Elsevier; Copyright 2014, JCBN; Copyright 2016, American Chemical Society.

- Structure: RNP, a polymeric micelle made of amphipathic block copolymers, can stably disperse in in vivo harsh conditions due to the entanglement of hydrophobic segments in its core (Figure 2a) [79]. It is reported that PEG imparts biocompatible characteristic to nanoparticle by inhibiting electrostatic and hydrophobic interactions with proteins and cells sterically, thereby increasing the stability of nanoparticle [80]. Unpaired radical in TEMPO is stable by preventing the coupling with each other due to the protection by four methyl groups surrounding it. However, since ROS are small molecular radical species, they rapidly react with TEMPO’s nitroxide radical. Although TEMPO is a highly reactive radical, it is conjugated via covalent linkage in the nanoparticle core; hence, non-specific interaction like LMW TEMPOL can be avoided upon administration. These characteristics potentially improve their accumulation in the target site via the EPR effect, which increases their therapeutic effects and prevents their premature renal excretion.
- Size: Core-shell type polymer micelles with several tens of nanometer in size (20–50 nm) ensures efficient accumulation in target intestinal mucosa (oral administration; colon cancer) or tumor vicinity (intravenous administration: breast cancer), additionally supported by the EPR effect [70,81,82]. It should be noted that the size range of RNP used in various anti-cancer studies, were small enough to prevent activation of the phagocytic system (≤100 nm cutoff size). Conversely, RNPs were large enough to evade rapid renal clearance (≥5.5 nm cutoff size) [83,84].
- Stability: Dynamic light scattering studies confirmed that RNP^O^ is stable under various pH 4–8.5, whereas pH-sensitive RNP^N^ was stable at pH 7.4 but decreased with a decrease in pH, confirming its collapse at low pH (diseased condition; tumor) (Figure 3a). Nonetheless, both the micelles maintained structural integrity at physiological pH 7.4, confirming the structural stability in the blood [77]. Furthermore, in ex vivo spiking experiments, we demonstrated that RNP do not internalize in the blood cells and prevents blood cell aggregation on the glass beads, which was in sharp contrast to TEMPOL (Figure 3b,c) [85]. This inert characteristic of RNPs with blood is extremely important for the systemic administration of nanoparticles.
- ESR active properties: ESR measurement shows a characteristics sharp triplet peak of the exposed TEMPO radical (an interaction between ^14^N nuclei and the unpaired electron), but when confined in the core of RNP, the ESR signal of TEMPO broadens at the same magnetic field, due to restricted mobility of the radicals in RNP’s solid core (Figure 2b) [75]. Due to this characteristic, it is very convenient to confirm the integrity and collapse of RNP and localization of RNPs for the pharmacokinetics studies.

RNPs have shown remarkable therapeutic effects with characteristics mentioned above than LMW antioxidant, TEMPOL, in various oxidative stress-related diseases such as cancer, colitis, cerebral hemorrhage, acute renal injury, Alzheimer’s disease and so on, attributed to their favorable pharmacokinetic properties [86].

### 4.2. Safety of RNPs

It was previously reported that TEMPOL induces apoptosis by impairing the oxidative phosphorylation and targeting complex I of the respiratory system affecting mitochondrial membrane potential in HL-60 cells [87]. In our studies, similar findings confirmed that LMW TEMPOL exerts adverse effects in various models, potentially caused due to its facile internalization into the normal cells and disruption of critical redox balance attributed to highly reactive nitroxide radicals. On the other hand, due to their higher molecular weight (ca. 10 kDa) and the self-assembling size (ca. 20–50 nm), RNPs avoid internalization into the normal cells and prevents disruption of their redox homeostasis [85,88]. For instance, as shown in Figure 3d, when zebrafish were maintained in 3 mM and 30 mM TEMPOL solution, they died within five days of TEMPOL addition. In contrast, in 30 mM RNP^O^-treated group, more than 95% of zebrafish survived at day 5, confirming low toxicity of RNPs. This safety was further confirmed by the negligible damage to zebrafish mitochondria in the RNP-treated group, while the elevated damage was observed in the TEMPOL-treated group (Figure 3e) [88]. In the ex vivo blood spiking experiment, we also confirmed that RNPs do not interact and internalize into the healthy blood cells (Figure 3c) and disrupt mitochondrial membrane potential of blood cells, which was in sharp contrast to TEMPOL [85]. In addition, the median lethal dose (LD_50_) of TEMPOL in C3H mice was 341 mg/kg through intravenous administration, whereas for RNP^N^, LD_50_ value was higher than 600 mg/kg (960 mmol N/kg) in ICR mice [77,89]. Extremely low toxicity of RNPs than LMW TEMPOL confirms that the confinement of conjugated TEMPO in the core of several tens of nanometer-sized self-assembled nanoparticles is necessary to avoid off-target effects and attain enhanced accumulation in the target tissue, leading to higher therapeutic effect.

### 4.3. Pharmacokinetic Properties of RNPs

**Figure 4:**
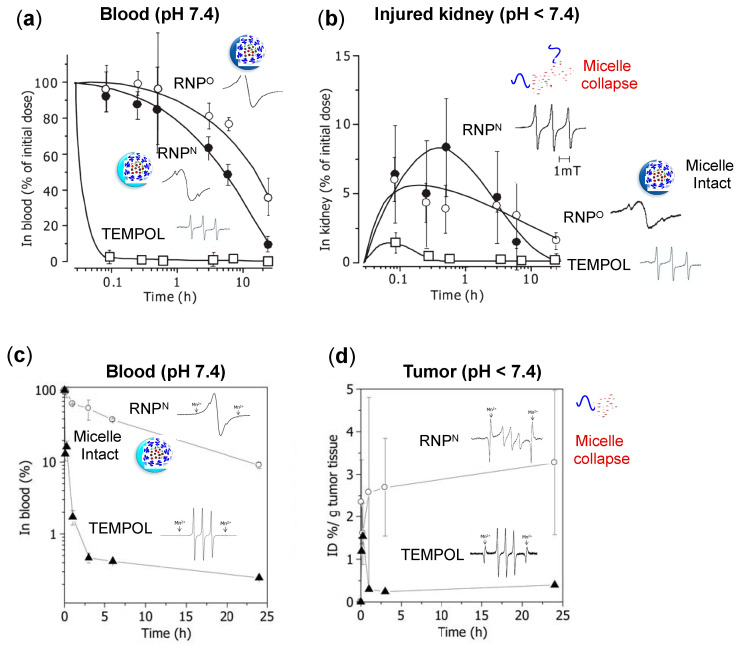
(**top**) Pharmacokinetic property of RNP^N^ and RNP^O^ in the blood and diseased organs. Time profile changes in the concentration of RNP^O^, RNP^N^, and TEMPOL; (**a**) blood and (**b**) injured kidney, after intravenous administration (75 mmol/kg of TEMPO concentration) in a renal ischemia-reperfusion induced acute kidney injury mice model [75,77]. The graph also displays ESR spectra of TEMPO radical of RNPs and TEMPOL. The ESR spectra of RNPs are broad under the physiological pH conditions (7.4), confirming their micelle integrity, in contrast with the sharp triplet peak of free TEMPOL. Under the decreased pH conditions, a typical diseased state, the pH-sensitive RNP^N^ group shows sharp triplet ESR signals, indicating the micelle collapse as compared to pH-insensitive RNP^O^, whose micelle integrity is unaffected. (**bottom**). Biodistribution of RNP^N^ and TEMPOL in a colon tumor (C-26 colon cancer cell line) bearing mice after intravenous administration with 40 mg/kg of TEMPO concentration; (**c**) blood and (**d**) tumor [82]. These data confirm that RNP^N^ is stable in the blood (broad ESR signal), while it is collapsed in the tumor environment due to the reduced pH (sharp ESR peaks). This figure is reproduced with permission from References [75,77,82]. Copyright 2011, Elsevier; Copyright 2014, WILEY-VCH Verlag GmbH & Co. KGaA, Weinheim; Copyright, 2013 Elsevier B.V.

TEMPOL, a low molecular weight compound with an exposed reactive nitroxide radical, has poor pharmacokinetics, which excretes rapidly after the administration. Thus, to suppress the rapid excretion and avoid the unwanted adverse effects, we covalently conjugated TEMPO to the amphiphilic block copolymer backbone with self-assembling characteristics, forming nanoparticle with several tends nanometer in size and accumulation tendency in the target inflamed site, such as a tumor. We confirmed this improvement of the pharmacokinetic property of RNPs in the renal ischemia-reperfusion induced acute kidney injury mice model [77]. In this study, an equal dose of RNP^O^, RNP^N^, and TEMPOL (TEMPO: 75 mmol/kg) were administered to ICR mice, after which TEMPO concentration was measured in the blood and kidneys by ESR. As shown in Figure 4a, TEMPOL cleared from the blood within 0.1 h of the administration, whereas RNP^O^ and RNP^N^ remained in the blood for more than 10 h. In the injured kidney (Figure 4b), TEMPOL was excreted within 0.5 h of administration, whereas RNP^O^ remained for 24 h and RNP^N^ managed to stay more than 10 h. This data confirmed that the kidneys, a major clearance organ, do not remove the RNPs as fast as LMW TEMPO due to their suitable structure and size. It is known that after reperfusion in the ischemic kidney, ROS level is significantly elevated, causing inflammation and decreased pH via acidosis [90]. As shown in Figure 4a, RNP^O^ and RNP^N^ in the blood are observed as intact micelles, assessed through a broader ESR signal than TEMPOL radical. In kidneys with acidic lesions, RNP^O^ integrity remains intact. In contrast, the ESR signal of RNP^N^ resembles to that of free TEMPOL radical (Figure 4b), implying the micelle collapse in response to acidic pH in the injured kidneys. This data confirms the pH sensitivity of RNPs in the diseased condition and their stability during systemic circulation compared to LMW TEMPOL.

We also confirmed the pharmacokinetics of RNP^N^ in a mice model of colon cancer, by intravenous administration of RNP^N^ and TEMPOL (40 mg/kg of TEMPO), which was assessed by ESR measurement [82]. As shown in Figure 4c, RNP^N^ remains in the blood even until 24 h (AUC, 769.49). In contrast, the LMW TEMPOL signal decreases drastically within 2 h (AUC, 19.2), which may be attributed to their diffusion into the normal cells and preferential renal clearance. The total accumulation of RNP in the tumor tissues (AUC, 39.6) was at least 6–7 fold higher than LMW-TEMPOL (AUC, 6.5). After 24 h of administration, RNP^N^ in tumor tissues was 8–9 fold higher (3.3% ID/g tumor tissue) compared to TEMPOL (0.4% ID/g tumor tissue) (Figure 4d). Interestingly, RNP^N^ remained intact as micelle in the blood and collapsed in a low pH tumor area, as assessed by ESR spectra. The reports mentioned above confirm that the covalent conjugation of TEMPO with amphiphilic copolymer and their self-assembling core-shell structure significantly suppresses their adverse effect and prolongs their presence in the systemic circulation. Due to the long blood circulation of RNP, they gradually accumulate in the target site via the EPR effect with negligible diffusion in the normal cells compared to LMW highly reactive TEMPO radical. With such favorable pharmacokinetics and negligible toxicity compared to LMW antioxidants, we evaluated the therapeutic efficacy of RNPs in various cancer models. The next section will discuss the application of RNPs in breast cancer, colon cancer, prostate cancer, and resistant epidermoid cancers as stand-alone or as adjuvants with conventional anti-cancer drugs.

### 4.4. RNPs for Cancer Therapy

#### 4.4.1. RNPs Inhibit the Tumorigenic Potential of Triple-Negative Breast Cancer

As stated above, we have succeeded in developing novel self-assembling antioxidants, which is less toxic and do not cause intracellular disturbance to the redox homeostasis of the normal cells. With these characteristics, the functionality of RNP as an anti-cancer drug was investigated in breast cancer. Breast cancer is the most common cancer occurring in women worldwide, with 2 million new cases diagnosed in 2018 (American Institute for Cancer Research). Due to the increase in the mortality rate of breast cancer patients, an alternative treatment is needed [91]. It was reported that breast cancer patients have significantly higher ROS levels such as superoxide and hydrogen peroxide in plasma, which correlated with the severity of the disease and altered antioxidant enzyme levels such as SOD in the tumor cells [92,93]. Copper, a potent oxidant, was also significantly elevated in the serum and tumor of cancer patients than the healthy subjects [94]. It is reported that copper induces HIF-1α and VEGF expression through the activation of the EGFR/ERK/c-fos transduction pathway promoting breast tumor angiogenesis and progression, which were reversed upon the addition of copper chelating agent and antioxidant NAC [95]. Menon et al. reported that the loss of redox control of the cell cycle might contribute to the aberrant proliferation of breast cancer cells [96]. Another report suggested that sublethal oxidative stress by H_2_O_2_/hypoxanthine and xanthine oxidase inhibited tumor cell adhesion to laminin and fibronectin and enhanced lung tumors of murine mammary carcinoma in an experimental metastasis model [97]. Based on these critical roles of oxidative stress in breast cancer survival, we evaluated the efficacy of our antioxidant self-assembled nanoparticle in a breast cancer model.

**Figure 5:**
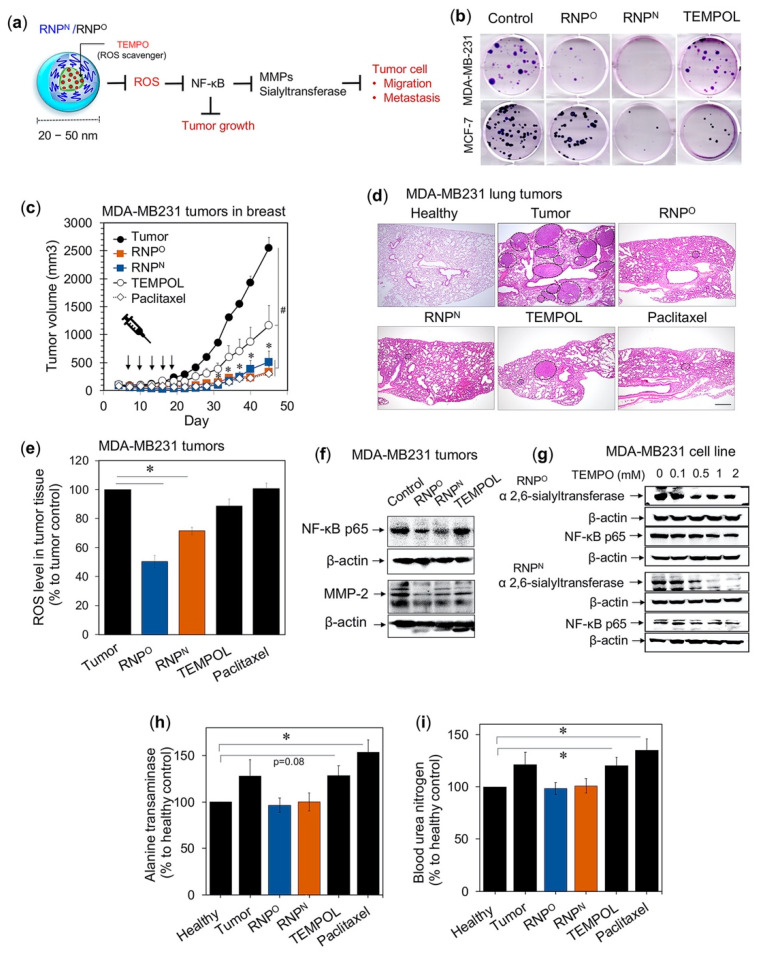
RNP^N^ and RNP^O^ inhibits the growth, proliferation, and metastatic potential of triple-negative breast cancer, MDA-MB231, with negligible adverse effects. (**a**) Schematic illustration of ROS-reduction mediated anti-tumor and anti-metastatic effect of RNPs. (**b**) Inhibition of colony-forming potential of breast cancer cell lines, MDA-MB231 and MCF-7 by RNP^N^, RNP^O^, and TEMPOL (IC_50_ value, 48 h). (**c**) Tumor growth profile of breast cancer cell line (MDA-MB231) in the mice xenograft model, intravenously administered with RNP^N^, RNP^O^, TEMPOL (74.13 mg/kg of TEMPO concentration), and conventional anti-cancer drug paclitaxel (10 mg/kg). (**d**) Representative histopathological H and E-stained lung sections of MDA-MB231 experimental metastasis model mice, intravenously administered with RNP^N^, RNP^O^, TEMPOL (18.5 mg/kg of TEMPO concentration) and paclitaxel (5 mg/kg). The encircled areas are representing MDA-MB231 tumors. Scale bar 500 μm. (**e**) ROS scavenging potential of RNPs in MDA-MB231 tumors detected by dihydroethidium compared to the untreated tumor as 100%. (**f**) Downregulation of MMP-2 and NF-kB expression by RNPs in MDA-MB231 tumors, as assessed by immunoblotting. (**g**) The ability of RNPs to suppress the expression of NF-kB and sialyltransferase, important enzymes assisting the metastasis of MDA-MB231 cell line, as assessed by immunoblotting [99]. Non-toxicity of RNPs as compared to LMW TEMPOL and paclitaxel after their intravenous administration, confirmed by liver and kidney damage markers: (**h**) alanine transaminase and (**i**) blood urea nitrogen, respectively. * *p* < 0.05 was considered significant [98]. This figure is reproduced with permission from References [98,99]. Copyright 2017, Elsevier Ltd.; Copyright 2018, Elsevier Ltd.

We investigated the anti-tumor and anti-metastatic effects of RNP^O^ and RNP^N^ using the triple-negative breast cancer cell line, MDA-MB231 (Figure 5a) [98,99]. Colony-forming assay was carried out in vitro using breast cancer cell lines, metastatic MDA-MB-231 and non-metastatic MCF-7. Treatment with IC_50_ values (RNP^O^; MDA-MB-231 = 2.20 mM, MCF-7 = 1.14 mM, RNP^N^; MDA-MB-231 = 3.00 mM, MCF-7 = 1.08 mM, and TEMPOL; MDA-MB-231 = 0.56 mM, MCF-7 = 0.46 mM), revealed that RNP^N^ showed the highest inhibition of colony-forming potential, followed by RNP^O^ and TEMPOL (Figure 5b). This data clearly indicates that the TEMPO-based antioxidants, RNP^N^ and RNP^O^, exerted a long-term inhibitory effect on the breast cancer cell growth regardless of their metastasis tendency than LMW TEMPOL with less toxicity. We next investigated in vivo efficacy of RNPs in a mouse xenograft model of breast cancer cell line, MDA-MB231. Intravenous administration of RNP^O^ and RNP^N^ (TEMPO; 74.13 mg/kg, five times, three days interval) showed a significantly decreased tumor growth than the untreated control and TEMPOL (Figure 5c). The tumor growth profile graph clearly shows that RNPs inhibit tumor growth much higher than TEMPOL and comparable to the conventional anti-cancer drug paclitaxel (10 mg/kg, five times, three days interval), indicating the importance of ROS scavenging in breast cancer treatments. We also confirmed that RNPs showed anti-metastatic effect by inhibiting the growth of MDA-MB231 lung tumors in an experimental metastasis model, which was higher than TEMPOL (TEMPO: 18.53 mg/kg/mouse, 10 times, 3 days interval) and comparable to paclitaxel (5 mg/kg/mouse, 10 times, 3 days interval) (Figure 5d). This decrease in tumor size exerted by RNPs corroborated with decreased tumor ROS, which was negligibly reduced in the TEMPOL-treated group (Figure 5e).

NF-kB is a redox-sensitive transcriptional factor which regulates expression of metallo-matrix protease (MMP-2) and α 2,6-sialyltransferae. MMPs function to degrade the extracellular matrix proteins and has been correlated with poor clinical outcome in breast cancer patients [10,100]. α 2,6-sialyltransferae catalyzes the addition of sialic acid to terminal oligosaccharides attached on the lipid or protein moieties of the tumor surface, which contributes to tumorigenesis, progression, and metastasis [101]. As shown in Figure 5f,g, both RNPs downregulate the expression of NF-kB, MMP-2, and α 2,6-sialyltransferae in MDA-MB231 tumors and cells, suggesting the mechanism of anti-tumor and anti-metastatic effect of RNPs. It should be noted that such high efficacy of our antioxidant nanoparticle was achieved with negligible adverse effects on the kidneys and livers, in contrast to LMW TEMPOL, and paclitaxel-treated group (Figure 5h,i). These reports suggest that our RNPs alone are more effective in inhibiting ROS-mediated tumorigenesis and metastasis of breast cancer as compared to LMW antioxidants.

#### 4.4.2. RNPs Inhibit the Tumor Growth and Progression of Colitis-Associated Cancer

**Figure 6:**
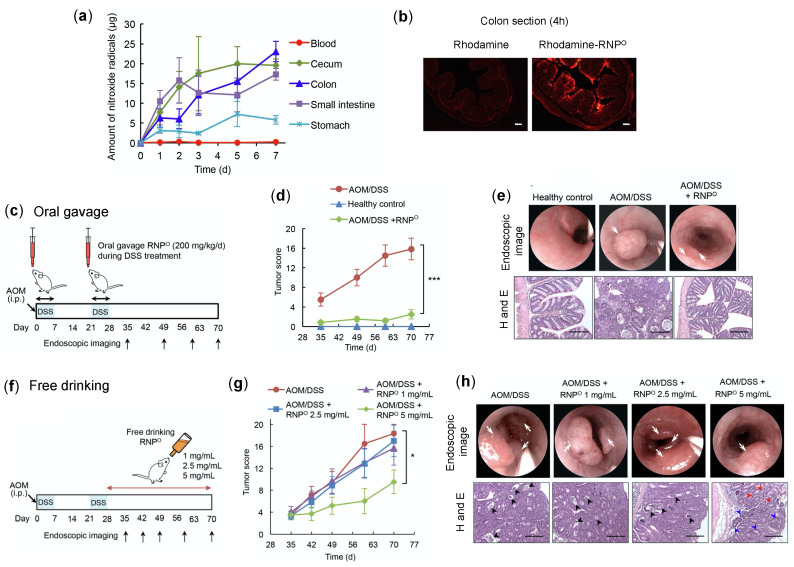
Anti-tumor effect of RNP^O^ in colitis-induced colon cancer (CAC) model. (**a**) Accumulation of RNP^O^ in the gastrointestinal tract by ad libitum drinking, assessed by ESR measurement [105]. (**b**) Localization of rhodamine-labeled RNP^O^ (rhodamine-RNP^O^) in the colon section, 4 h after the oral administration with 5 mg/mL of rhodamine-RNP^O^ (1 mL), scale bar 200 μm [106]. (**c**) The scheme showing anti-tumor effect (protective) of orally administered RNP^O^ in azoxymethane (AOM) and dextran sodium sulfate (DSS) (AOM/CAC) induced colitis-associated cancer in mice. RNP^O^ (200 mg/kg/d) was administered by oral gavage during the two weeks of the DSS treatment period. (**d**) RNP^O^ inhibits the formation of colon tumor, confirmed by tumor score and assessed by endoscopy. (**e**) The endoscopic imaging of mice colon, displaying tumor shown by white arrows and H and E-stained colon tissues (scale bar 100 μm) at the experimental endpoint (day 70). (**f**) The scheme showing anti-tumor effect (therapeutic) of ad libitum drinking of RNP^O^ in AOM/CAC-induced colitis-associated cancer in mice. RNP^O^ (1, 2.5, and 5 mg/mL) was available as ad libitum drinking after AOM/DSS treatment. (**g**) The therapeutic effect of RNP^O^ to inhibit the formation of colon tumor as confirmed by tumor score, which was assessed by endoscopy. (**h**) The endoscopic imaging of mice colon, displaying tumor shown by white arrows and H and E stained colon tissues (scale bar 100 μm), at the experimental endpoint (day 70). Black arrows in H and E colon stained tissues indicate the necrotic cells surrounded by cancer cells, blue arrows indicate adenoma, and red arrows display normal crypts [105]. * *p* < 0.05 was considered significant. This figure is reproduced with permission from References [105,106]. Copyright 2018, Elsevier Ltd.; Copyright 2012, AGA Institute (Elsevier publisher).

An increase in oxidative stress and oxidative cellular damage promoting carcinogenesis has been observed in inflammatory bowel disease patients [102,103]. Ulcerative colitis (an inflammatory bowel disease) associated with colon cancer (CAC) is the third most common malignancy and one of the major causes of cancer-related death [104]. With these facts, we tested the efficacy of RNPs to suppress the oxidative stress-mediated tumor formation in the mice colon [105]. The pharmacokinetics of RNP^O^ by free drinking confirmed that RNP^O^ accumulates in the colon with negligible internalization in the blood (ESR measurement) (Figure 6a). The localization of rhodamine-labeled RNP^O^ further validated the colon accumulation of RNP^O^ after 4 h of administration. As shown in Figure 6b, the rhodamine-labeled RNP^O^ was strongly observed in the colon, especially in the colon’s mucosa area. In contrast, no fluorescent was observed in rhodamine administered group, as it was excreted out sooner than RNP^O^. This data confirmed that LMW antioxidants might not be suitable for CAC treatment due to their poor retentivity in the colon [106]. The effect of RNPs on colon cancer was investigated by a CAC mice model, which was prepared by intraperitoneal injection with azoxymethane (AOM) (10 mg/kg body weight) followed by two cycles of 7d-treatment of 3% dextran sodium sulfate (DSS) (Figure 6c). As shown in Figure 6d,e, the oral administration of RNP^O^ during DSS treatment significantly suppressed the tumor formation in the colon, which was confirmed by endoscopy and H and E stained colon tissues. In the RNP-treated group, no change in the body weight was observed compared to AOM/DSS control, which was significantly reduced during DSS treatment (data not shown).

It is worth noting that such an effect of RNP^O^ was supported with decreased colitis disease index and pro-inflammatory cytokine interferon-gamma (IFN-γ). These results indicate the potential of RNP^O^ to reduce colitis-induced inflammation, which is a major factor for the induction of colon cancer. Ad libitum drinking of RNP^O^ solution (5 mg/mL) after AOM and DSS treatment also significantly suppressed the tumor formation in the colon as assessed by endoscopy and histology (Figure 6f–h). These reports confirmed that RNP^O^ is an effective and suitable nano-antioxidant for the treatment of colon cancer.

#### 4.4.3. Synergistic Effects of RNP and Fibrinolytic Tissue Plasminogen Activator for Colon Cancer Therapy

It is well known that the efficacy of drugs to inhibit tumor growth depends on whether the drug has sufficiently reached the target site or not, which depends on the blood perfusion status within the tumor vessels [107]. The tumor microenvironment is complex, comprising of extracellular matrix (ECM) components such as fibrin, elastin, laminin, collagen, platelet aggregation, etc. The ECM is known to obstruct the blood flow and perfusion to the tumor areas, limiting the effective delivery of drugs, contributing to inadequate drug response, and promoting drug resistance [108].

Degradation of ECM components from the tumor environment is a robust strategy to improve vascularization and blood supply to the tumors. Fibrinolytic tissue plasminogen activator (t-PA), is a member of the serine protease family, physiologically involved in the matrix regulation and homeostasis of the blood coagulation/fibrinolysis [109]. Zhang et al. reported on the use of t-PA for modulating the tumor microenvironment to improve the delivery efficiency of anti-cancer drug to the target site [110].

**Figure 7:**
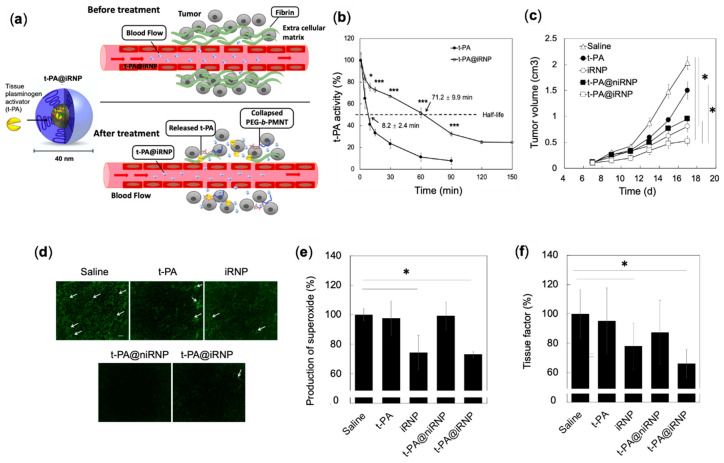
Anti-tumor effect of fibrinolytic tissue plasminogen activator installed in radical containing nanoparticles (t-PA@iRNP). (**a**) Schematic illustration of the delivery and therapeutic effect of t-PA@iRNP in tumors characterized by dense fibrin extracellular matrix [112]. (**b**) Ex vivo thrombolytic activity of t-PA enzyme, after intravenous administration in mice with equimolar dose of naked t-PA and t-PA@iRNP, measured using t-PA’s ability to hydrolyze a tri-peptide chromogenic substrates of H-d-isoleucyl-l-prolyl-l-arginine-p-nitroanilide dihydrochloride to p-nitroaniline. Liberated p-nitroaniline was measured spectrophotometrically at 405 nm by using a UV-Vis spectrometer [113]. (**c**) Tumor growth profile in a C-26 colon murine cancer model, intravenously administered (5 times) with saline (control), t-PA (0.04 mM), and iRNP (TEMPO; 5.3 mM). (**d**) Representative images of fibrin immunofluorescence (white arrow) in the tumor tissues, scale bar 10 μm. (**e**) Superoxide level in tumor tissue homogenate measured by ROS sensitive dye, dihydroethidium. (**f**) Tissue factor in tumor lysates measured by ELISA [112]. * *p* < 0.05 was considered significant. This figure is reproduced with permission from References [112,113]. Copyright 2020, Elsevier Ltd.; Copyright 2019, Elsevier Ltd.

Because the half-life of the naked t-PA is extremely short (<5 min), continuous and invasive intravenous administrations are required to show their effectiveness [111]. In this line, we employed RNP as a new delivery platform for t-PA, which not only acts as DDS with favorable pharmacokinetics but also contributes to the anti-tumor effect through ROS scavenging characteristic (Figure 7a) [112,113]. t-PA@iRNP (hereafter “i” in iRNP denotes the core composed of polyion complex) is a core-shell structured polyion complex (PIC) micelle consisting of three components: (i) ROS scavenging cationic PEG-*b*-PMNT diblock amphiphilic copolymers, (ii) anionic poly (acrylic acid) (PAAc) and (iii) fibrinolytic t-PA (Figure 7a). We found that t-PA@iRNP retained their enzymatic activity after 2 h (t_1/2_ = 71 min) of intravenous administration, whereas the activity of naked t-PA decreased within 0.5 h (t_1/2_ = 8 min) (Figure 7b) [113]. The prolonged enzymatic activity of t-PA@iRNP than naked t-PA is due to the stable encapsulation of t-PA in the iRNP matrix, which protected it from the enzymatic degradation. We have previously confirmed that t-PA@iRNP had almost no enzymatic activity under the physiological pH (7.4). On the other hand, upon decreasing the pH, its enzymatic activity was significantly increased, indicating the pH responsive collapse of t-PA@iRNP. Intravenous administration of t-PA@iRNP (t-PA; 0.04mM and iRNP; 5.3 mM TEMPO, five times with interval of three days) to mouse xenograft model of C-26 colon cancer cell line, showed effective suppression of tumor growth as compared to control, t-PA@niRNP (no antioxidant capacity), naked t-PA, and iRNP, validating the synergistic effect of iRNP and t-PA (Figure 7c) [112]. Interestingly, iRNP alone also showed a significant anti-tumor effect on colon tumors. It should be noted that the pharmacokinetics of t-PA upon encapsulation by iRNP is favorably changed for in vivo application. This pattern can be seen in t-PA@niRNP, where a higher effect of t-PA could be observed when encapsulated in niRNP (no antioxidant capacity), than the naked t-PA itself, indicating the importance of delivery systems for the proteins (Figure 7c,d).

We also confirmed that the higher effect of t-PA@iRNP was due to higher fibrin degradation in the tumor area by t-PA (Figure 7d), decreased ROS (Figure 7e), and downregulated NF-kB by iRNP (data not shown). Both t-PA@iRNP and iRNP-treated group significantly reduced the expression of ROS-regulated tissue factor, which activates coagulation and platelets essential for tumor growth and metastasis increase (Figure 7f) [114,115]. Based on these results, it is clear that RNP possesses bidentate roles, viz., effective carriers for t-PA to target solid tumors and suitable anti-cancer drugs, which effectively scavenge overproduced ROS around the tumor environment.

#### 4.4.4. RNPs Enhances the Therapeutic Efficiency of Pioglitazone on Prostate Cancer

**Figure 8:**
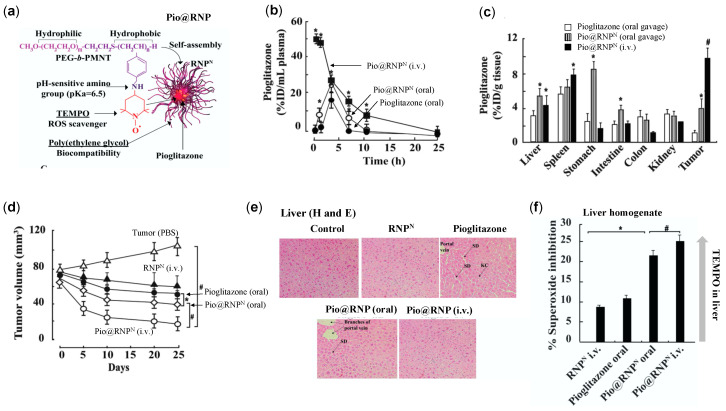
RNP increases the chemotherapeutic efficiency of pioglitazone (Pio) and suppresses its adverse effects. (**a**) Chemical structure of RNP^N^’s polymer (PEG-b-PMNT) and illustration of pioglitazone encapsulated in RNP^N^. (**b**) Systemic bioavailability pioglitazone (oral), Pio@RNP^N^ (oral), and Pio@ RNP^N^ (i.v.) in mice administered with 15 mg/kg of pioglitazone. (**c**) Biodistribution of pioglitazone in various organs of mice after the treatment period of 25 days. (**d**) Tumor growth profile of prostate cancer (PC-3) in a mouse model, administered with PBS (control), free pioglitazone and Pio@ RNP^N^ (Pio: 15 mg/kg and RNP^N^: 300 mg/kg). (**e**) RNP^N^ suppresses the adverse effect exerted by pioglitazone as assessed by liver histology stained by H and E (SD: sinusoidal dilatation, KC: Kupffer cells). (**f**) Superoxide inhibitory activity in liver homogenates administered with samples (from tumor xenograft studies) as evaluated by xanthine-xanthine oxidase assay [116]. * *p* < 0.05 was considered significant. This figure is reproduced with permission from Reference [116]. Copyright 2016, Elsevier Ltd.

Pioglitazone belongs to thiazolidinediones family that shows efficacy in type 2 diabetes mellitus and cancer, accompanied by several adverse effects such as hepatotoxicity, cardiac abnormalities, and weight gain due to fluid retention [116]. In addition to severe toxicity exerted by pioglitazone, poor solubility and low bioavailability due to extensive liver metabolism are also its drawbacks [117]. Several reports have confirmed that liver metabolism of pioglitazone forms reactive oxidative intermediates that potentially damages hepatocytes [118]. Considering this, we prepared RNP encapsulated with pioglitazone (Pio@RNP^N^) to prevent premature metabolism of pioglitazone in the liver by modulating its pharmacokinetics property and decrease its ROS-mediated adverse effect by TEMPO radical of RNP (Figure 8a) [116]. Pharmacokinetic studies revealed that oral administration of Pio@RNP^N^, enhanced systemic presence of pioglitazone (AUC: 113.2) to twice as compared to free pioglitazone (oral) (AUC: 51.2), whereas intravenous administration Pio@RNP^N^ showed the highest plasma concentration of pioglitazone (AUC: 723.9) (Figure 8b). In this study, free pioglitazone was administered orally in a CMC formulation due to its low solubility, whereas no such issue was observed in the encapsulation of pioglitazone in RNP. Biodistribution studies confirmed that Pio@RNP^N^ (intravenous; i.v.) accumulated highest in tumor tissues (10% ID/g tissue) followed by oral administration of Pio@RNP^N^ (3.8% ID/g tissue) and oral pioglitazone (1.2% ID/g tissue) (Figure 8c).

With such favorable pharmacokinetic properties of Pio@RNP^N^ over free pioglitazone, its anti-cancer therapeutic efficacy was tested in a mouse xenograft model of prostate cancer (PC-3) (Figure 8d). At the experimental endpoint, orally administered pioglitazone reduced tumor volume by only 25%, Pio@RNP^N^ (oral) by 36%, while Pio@RNP^N^ (i.v.) showed the highest anti-tumor effect with 60% growth inhibition. In addition, intravenous administration of Pio@RNP^N^ largely protected the liver toxicity exerted by ROS induced by the pioglitazone treatment (Figure 8e). An ex vivo xanthine/xanthine oxidase (superoxide scavenging) assay was conducted to measure the ROS scavenging effect of the TEMPO in the liver homogenates of the treated mice. In this assay, the xanthine/xanthine oxidase system generates superoxide ion radicals detected by nitro blue tetrazolium. When liver homogenates from treated mice are spiked with xanthine/xanthine oxidase, the generated superoxide ions are scavenged by antioxidants in liver homogenates, in our case, nitroxide radical (TEMPO). Higher the TEMPO in the liver homogenates, the higher the superoxide inhibition/scavenging ability. Figure 8f shows that Pio@RNP^N^ (i.v.) exerts highest superoxide ion inhibition potential than orally administered Pio@RNP^N^, and pioglitazone itself, suggesting the localization of RNP^N^ in liver which might have contributed to the inhibition of pioglitazone-mediated adverse effect. This data corroborated with the result of lipid peroxidation status in the liver. We confirmed that Pio@RNP^N^ (i.v.) treated group had a significantly lower lipid peroxidation level than pioglitazone (data not shown). These reports highlights the potential of RNP^N^ as a DDS that increases the therapeutic efficacy of pioglitazone and decreases its adverse effects.

#### 4.4.5. RNPs Enhances the Therapeutic Efficiency of Doxorubicin on Colon Cancer and Epidermoid Cancers

The effectiveness of cancer chemotherapy is greatly limited due to the drug-resistant characteristics of tumor cells, attributed largely to their drug efflux system [119]. It is reported that P-glycoprotein (P-gp) and multi-drug resistance-associated protein-1 (MRP1), which belong to the ATP-binding cassette (ABC) transporter superfamily, are overexpressed in various cancers [120]. P-gp and MRP-1 both have been reported to confer resistance to various cancers against anti-cancer drugs [121]. With this fact in mind, several drug combination approaches have been applied that use ABC transporter inhibitors as adjuvants to overcome the drug resistance and potentiate the anti-cancer drug efficacy. For instance, administration of dofequidar, a P-gp inhibitor, with anti-cancer drugs such as cyclophosphamide, doxorubicin (Dox) and fluorouracil to patients with advanced or recurrent breast cancer, increased progression-free survival days from 241 (without P-gp inhibitor) to 366 [122]. However, several clinical trials have largely failed to manifest the therapeutic efficacy of such anti-cancer drugs/adjuvants. For instance, no improvement in the disease-free survival was observed in recurring or refractory multiple myeloma patients with and without P-gp inhibitor, valspodar, in conjunction with vincristine, Dox, and dexamethasone [123]. Although drug-efflux system inhibition for multi-drug resistance tumor therapy seems to be robust, these effects are not noteworthy. Binkhathlan et al. attributed the apparent failure of these adjuvants (drug efflux inhibitors) to demonstrate clinical efficacy to their non-specific action and distribution, causing toxicities due to their LMW [124]. Another possibility might be that the inhibitors themselves were not compelling enough. In this line, improved drug efflux inhibitors or the use of the delivery platform that specifically accumulates in the resistant tumors are highly desirable.

It was reported previously that oxidative stress is strongly related to this drug resistance. For example, ROS activates NF-kB, which increases drug efflux proteins such as P-gp and MRP-1, located in the cellular membrane [125]. It was also previously reported that P-gp and MRP-1 both are regulated independently by ROS in cancers [126,127]. Therefore, antioxidants are one of the candidates to suppress this drug resistance and increase the efficacy of anti-cancer drugs. Although pre-administration of LMW antioxidants such as edaravone and TEMPO have been evaluated to suppress the drug resistance of cancers, the results are not satisfactory [60,128]. Despite the fact that the antioxidant application for the chemoresistant cancer treatment may be in the right direction, however, they might not be effective due to the preferential clearance properties or low systemic retention as stated above. Therefore, in this line, we applied antioxidant RNPs to overcome the shortcoming of LMW drug efflux protein inhibitors/antioxidants by decreasing the ROS associated drug resistance.

**Figure 9:**
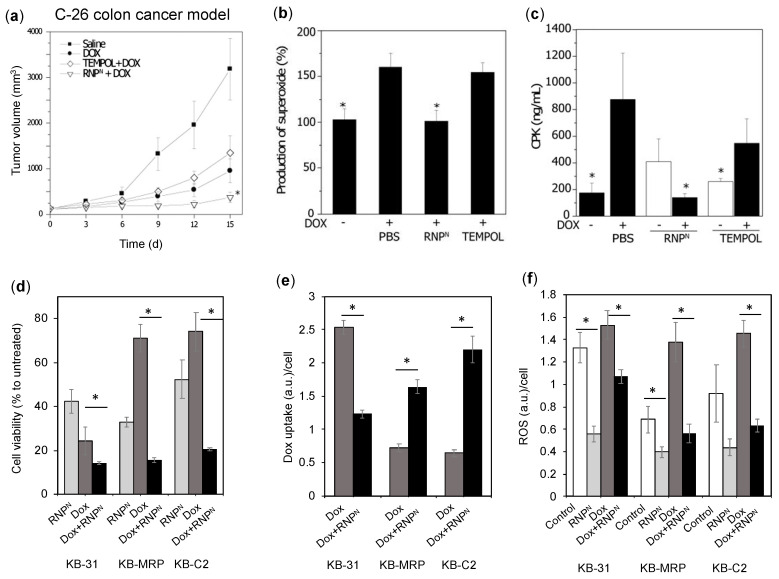
**(top)**. RNP increases the therapeutic effect of doxorubicin (Dox) in a colon cancer model. (**a**) Tumor growth profile of subcutaneous colon tumor (C-26) pre-treated with RNP^N^ (i.v., 100 mg/day for 4 days, days−4 to −1), followed by a single injection of DOX (i.v., 10 mg/kg on day 0). The ability of RNP^N^ to inhibit the cardiotoxicity of Dox mediated by increased ROS [82]; (**b**) inhibition of superoxide level by RNP^N^ in the heart homogenates, (**c**) creatine phosphokinase (CPK) in plasma, a marker of heart damage. Mice were intravenously injected with RNP^N^ (25 mg/kg/day) and LMW-TEMPOL (4 mg/kg/day), followed by DOX (20 mg/kg, i.v.) 30 min later. 3 days post Dox administration, samples were analyzed. (**bottom**). RNPs increases the therapeutic effect of Dox by overcoming drug resistance in the epidermoid cancer cell lines. (**d**) Cytotoxicity of combination treatments: RNP^N^ (2 mg/mL) and Dox (5 μg/mL) for 48 h in epidermoid cancer cell lines-drug sensitive KB-31, drug-resistant KB-MRP overexpressing drug efflux transporter, MRP-1 and drug-resistant KB-C2 overexpressing drug efflux transporter, P-gp; (**e**) Dox uptake in epidermoid cancer cell lines after 2 h of treatment (RNP^N^ (2 mg/mL) and Dox (5 μg/mL)). (**f**) ROS level after 24 h of treatment with RNP^N^ (2 mg/mL) and Dox (5 μg/mL) in epidermoid cancer cell lines [130]. * *p* < 0.05 was considered significant. This figure is reproduced with permission from Reference [82] and adapted from [130]. Copyright 2013, Elsevier B.V.; Copyright 2017, Elsevier Ltd.

Dox, is known to generate ROS in vivo, which results in severe adverse effects in the normal tissues and increases the drug resistance of tumors [129]. Thus, we evaluated the ability of our RNPs to sensitize cancer cells and potentiate the efficacy of Dox by scavenging ROS in the colon and epidermoid cancer models. We have previously shown that intravenous administration of RNP accumulates significantly higher in C-26 colon tumors, while LMW TEMPOL excretes faster (Figure 4c,d) [82]. To confirm the sensitizing effect of RNPs, in a C-26 colon cancer model, we pre-administered RNP^N^ (i.v.) for 4 days, followed by Dox administration (10 mg/kg) [82]. The RNP^N^ + Dox-treated group showed the highest tumor growth suppression, followed by the free Dox administration group, as shown in the tumor growth profile graph (Figure 9a). It is interesting to see that pre-administration of TEMPOL did not decrease any tumor growth at all as compared to Dox alone, which indicates the poor systemic and tumor presence of TEMPOL compared to RNP^N^ (Figure 4c,d and Figure 9a). As previously mentioned, Dox increases the ROS, which is one of the reasons for its off-target effects on the heart and several other organs. We confirmed that pre-administration of RNP^N^ decreases the ROS in the heart tissues (Figure 9b), which prevented Dox-induced cardiotoxicity as assessed by creatine phosphokinase, a marker for myocardial damage (Figure 9c). Such protective effect was not seen in the TEMPOL treated group. These data implied that RNP not only potentiate the efficacy of Dox against colon cancer but also decreases its adverse effects.

In our next study, we confirmed this effect by co-treatment of RNP^N^ with Dox in 3 different types of epidermoid cancer cells: Drug-sensitive KB-31, drug-resistant KB-C2 (overexpressing P-gp) and KB-MRP (overexpressing MRP-1) (Figure 9d–f) [130]. As shown in Figure 9d, the viability of resistant cancer cell lines with the combination treatment of RNP^N^ + Dox decreases significantly as compared to the Dox alone (48 h treatment). These significantly different efficiencies corroborated with Dox uptake tendencies; where in RNP^N^ treatment (2 h), a significantly higher Dox uptake was observed in contrast to cells without RNP^N^ (Figure 9e). Figure 9f shows that ROS is elevated in the resistant cancer cells that may further confer resistance to the cancer cells, which was significantly reduced upon RNP^N^ treatment. It should be noted that the drug-sensitive cell line, KB-31, was sensitive to RNP and Dox treatment, with high internalization of Dox, confirming negligible drug resistance level due to low drug efflux proteins. These data imply that the antioxidant activity of RNP is essential to modulate the drug efflux proteins by scavenging regulatory ROS, allowing the enhanced internalization and toxicity of Dox. Based on these data, it is concluded that RNP is a potential antioxidant to decrease the drug resistance of various cancers.

## 5. Conclusions

Cancers are characterized by persistent elevated intracellular ROS, critical for their survival, proliferation, angiogenesis, and metastasis. Therefore, the use of antioxidants is a suitable choice of therapeutic interventions to impede tumorigenesis. However, the failure of LMW antioxidants to inhibit tumors clinically accentuates the need for new therapeutic strategies to limit various cancers. In this line, our newly developed self-assembling antioxidants, RNP^O^ and RNP^N^, both have shown effective ROS-reduction mediated anti-cancer effect in vitro and in vivo as stand-alone or as an adjuvant to reduce aggressiveness and/or sensitize several cancers for chemotherapy. Higher bioavailability, specific tumor accumulation, and negligible toxicity of RNPs make them more suitable antioxidant therapeutic intervention than LMW counterparts for the cancer treatment. Recently, several other groups have started antioxidant therapy based on their own design. For instance, Moriyama et al., prepared antioxidant micelles from poly (ethylene glycol)-b-poly (dopamine) block copolymers that inhibits angiogenesis in the chicken ex ovo chorioallantoic membrane assay [131]. Rocha et al., also developed epigallocatechin-3-gallate incorporated polysaccharide nanoparticles which inhibited Du145 prostate cancer cells in vitro [132]. Including their work, the authors hope to establish a new field for antioxidant-based cancer therapeutics.

## Data Availability

Not applicable.
